# Residual characteristics of etofenprox in the processing stages of rice cakes and cookies

**DOI:** 10.1371/journal.pone.0255751

**Published:** 2021-08-06

**Authors:** HyeSu Lee, Moo-Hyeog Im

**Affiliations:** Department of Food Engineering, University of Daegu, Gyenogsan, Gyeongbuk, Korea; Cairo University, EGYPT

## Abstract

The changes in residual amounts of an insecticide (etofenprox) in processed rice cakes and cookies were investigated in this study. Test samples were sprayed with etofenprox during rice cultivation, and brown rice samples were dipped in a pesticide solution to investigate the effects of washing and processing. A multiresidue method for multiclass pesticides was employed for etofenprox analysis using a high-performance liquid chromatography–ultraviolet detector setup. Etofenprox was not detected in polished rice that was processed into rice cakes and cookies. The etofenprox residue levels were 2.13 mg/kg in each processing stage of brown rice products that were dipped in 400 mg/kg etofenprox solutions. The residual amounts of etofenprox in washed/polished rice and rice flour obtained by grinding were 1.25 and 0.77 mg/kg, respectively. The residual levels were 0.38 mg/kg in rice cakes prepared by cooking rice flour in a steamer for 20 min (a decrease of 82.1% compared to that in polished rice), 0.47 mg/kg in rice cookies baked in an oven for 20 min (a decrease of 78.0%), and 0.21 mg/kg in fried rice cookies (a decrease of 90.2%). Overall, the residual levels of etofenprox decreased in a range of 40–100% during the processing of rice cakes and cookies.

## Introduction

Rice is a major grain and plays a significant role in the human diet; it is consumed as a staple food by more than half of the global population, and thus, is an important part of plans that aim to secure basic food resources for poverty eradication and dealing with undernutrition [1, 2]. Pesticides are an important part of rice production; they contribute to the prevention and control of pests and diseases, as well as improvement in quality during the cultivation and storage of rice and other agricultural products [3]. In addition, pesticides play an important role in reducing agricultural labor and production costs, and therefore, contribute significantly toward the improvement of the lives of farmers [4]. However, most pesticides are not found in nature and are chemically synthesized as organic compounds; these pesticides are harmful to human health as their residues in agricultural products can lead to chronic toxicity. Therefore, problems involving pesticide residues have been widely discussed [3–5].

Most of the pesticide residues in agricultural raw materials are removed by several methods during processing and cooking, such as washing, thermal decomposition, and volatilization. However, in some cases, pesticide residue levels increase compared to those in the raw materials during drying or concentration processes [6, 7].

As rice-based processed foods have become more convenient and diversified, various research-based efforts have been undertaken to develop more diverse processed rice products [8, 9]. Previous studies on processed rice products have focused on, diverse topics such as the quality characteristics of such products [10–16]. The lack of research on the reduction rate of pesticide residues in the stages involved in rice processing necessitates further investigation in this regard; this can facilitate the risk assessment of foods that is typically carried out via maximum residue limits (MRLs) for pesticides [17].

Etofenprox is a synthetic pyrethroid insecticide that can simultaneously prevent and control various pests, such as planthoppers [18]. Etofenprox is primarily metabolized by desethylation of the ethoxyphenyl group, hydroxylation of the phenoxy ring, and oxidation of the benzyl moiety with subsequent cleavage of ether linkages to form polar compounds [19]. Reports published by the Joint Meeting on Pesticide Residues (JMPR) provide information on the acceptable daily intake (ADI) values of etofenprox, which are 0–0.03 mg/kg according to the Codex Alimentarius (Codex) guidelines, and 0.03 and 0.031 mg/kg according to the South Korean and Australian, and Japanese guidelines, respectively [19]. The no-observed-adverse-effect level (NOAEL) is 3.1 mg/kg·bw according to the Australian guidelines [19]. The JMPR also submits reports on the metabolites and toxicology of pesticide residues. The half-life of etofenprox in rice is two weeks, and 90% of the original etofenprox is degraded after 20 d. In animals, etofenprox was found to be rapidly excreted when provided to male and female rats, which excreted more than 80% of the administered dose within 48 h through feces and urine, with the major route being via feces [19].

Previous studies on etofenprox have focused on several topics regarding residue levels, such as the biological half-life of pesticide residues in Chinese cabbages and changes in pesticide residues over specific durations [20], the effect of drying on the processing factors of pesticide residues in Chinese matrimony vines [21], and on the levels of pesticide residues in brown rice and polished rice samples sprayed with etofenprox [22]. The JMPR submits reports that establish the Codex MRLs for pesticide residues. Among these, studies on etofenprox residues have been limited to establishing MRLs in raw agricultural products such as rice, citrus fruits, pome fruits, peaches, and plums; however, changes in the pesticide residue levels in processed rice products have not been investigated to date [23]. Therefore, etofenprox was selected for investigation in the present study because the residual patterns of etofenprox during rice processing have not yet been systematically studied, although the MRL for etofenprox has been established in the Korea Food Code and extensively employed in rice cultivation.

This study was aimed at investigating the residual characteristics of etofenprox during the processing stages of rice-based products. Rice was harvested after being sprayed with a dose of etofenprox that was three times higher than the recommended safe dose for rice cultivation. In addition, the reduction rates of pesticide residue levels were examined using samples of brown rice dipped in etofenprox solutions to accurately probe the residual characteristics of etofenprox during rice processing.

## Materials and methods

### Materials

The etofenprox analytical standard employed in this study was procured from Dr. Ehrenstorfer GmbH (99.0% pure; Ausgburg, Germany). Acetone, n-hexane, and dichloromethane were used for extraction and cleanup of pesticide residues and were purchased as guaranteed reagent-grade products from J.T. Baker (Center Valley, Pennsylvania, USA). Acetonitrile (purchased from J.T. Baker) was used as a solvent in high-performance liquid chromatography (HPLC). Florisil was sourced from Sigma-Aldrich Chemie (St Louis, Missouri, USA) and used as an adsorbent for open column chromatography. Sodium sulfate anhydrous was obtained from Junsei Chemical (Tokyo, Japan) and employed as a drying agent.

### Pesticide application

Pesticide was sprayed in an experimental field (area: 156 m^2^) in Anseong, Gyeonggi Province (Republic of Korea). Etofenprox 10% EC (Bisangtan®, Kungnong, Seoul, Republic of Korea) was used in the form of a spray solution at a concentration three times higher (48.0 g ai/10a) than that recommended for the safe use of the pesticide (16 g ai/10a). The foliar spraying method was used to apply etofenprox three times at intervals of nine days. This protocol was followed in accordance with guidelines published by the Food and Agricultural Organization (FAO) [24] for investigating pesticide reduction rates during rice processing. If the pesticide is sprayed at the recommended safe dose, it is removed after processing and the reduction rate cannot be determined; therefore, a higher pesticide dose was employed. Moreover, an excessive amount of the pesticide can lead to phytotoxicity in the rice. Therefore, a dose three times higher than that recommended for the safe use of the pesticide was adopted to ensure detectable levels of pesticide residuals in brown rice and avoid phytotoxicity. Rice was harvested 14 d after the final pesticide application and subsequently dried to yield rice grains. The etofenprox residue level in samples of rice sprayed with pesticides during cultivation (RSPC) was 0.21 mg/kg in brown rice. The residual amount of etofenprox in polished rice, which was obtained by milling of brown rice, was 0.04 mg/kg; the polished rice grains were subsequently used as samples for the preparation of processed rice foods.

In the case of the pesticide sprayed during cultivation, determining the tendency of reduction in residue levels after processing was expectedly difficult owing to the low residue levels of the pesticide. Therefore, the reduction rates of the residue levels of etofenprox were examined using samples of brown rice dipped in etofenprox solutions to accurately probe its residual characteristics during rice processing. Additionally, this investigation can assist in determining the reduction rate of the pesticide if it is accidentally over-sprayed during the actual cultivation process. Etofenprox solutions with concentrations of 15 and 400 mg/kg were prepared using commercially available etofenprox 10% EC (Bisangtan®). The brown rice samples were dipped in these etofenprox solutions for a short duration (10 s) to ensure pesticide residue levels of 0.78 mg/kg in rice dipped in etofenprox at the MRL (RD 1) and 31 mg/kg in rice dipped in etofenprox at 30 times the MRL (RD 30). The results of the analysis of etofenprox residues and MRL of etofenprox are listed in Table 1 [25]. These samples were subsequently dried to a moisture content of 13–15% at 30°C. The residual amount of etofenprox in RD 1 after the milling of brown rice into polished rice was 0.10 mg/kg, whereas that of RD 30 was 2.13 mg/kg. The polished rice was subsequently used for rice processing analysis.

**Table 1:** Residual characteristics of brown rice and polished rice (n = 3).

Sample	Etofenprox residual levels (mg/kg) Mean ± SD		MRL^a^ of etofenprox (mg/kg)
	Brown rice	Polished rice	
**RSPC**^b^	0.21 ± 0.013	0.04 ± 0.001	1.0^e^
**RD 1**^c^	0.78 ± 0.02	0.10 ± 0.005	
**RD 30**^d^	31.1 ± 1.07	2.13 ± 0.05	

^a^MRL: maximum residue level

^b^RSPC: samples of rice sprayed with pesticides during cultivation

^c^RD 1: samples of brown rice dipped in an etofenprox solution at the MRL

^d^RD 30: samples of brown rice dipped in an etofenprox solution at a residue level 30 times higher than the MRL.

^**e**^1.0: Pesticide MRLs in agricultural commodities in the Republic of Korea [25]

### Residue analysis

The pesticide residues in the RSPC, RD 1, and RD 30 samples were analyzed following the second protocol described the multiresidue methods for multiclass pesticides in the Korea Food Code [26]. Moreover, the cartridge cleanup method was modified and employed. The experimental protocol involved in the analysis of pesticide residues is described below.

Deionized water (30 mL) was added to 50 g of the sample, and the resulting moist sample was allowed to sit for 1 h. Acetonitrile (100 mL) was subsequently added to this mixture, followed by high-speed grinding and extraction for 3 min at 14,000 rpm. The extract was flowed through celite 545 to facilitate suction filtration, and the filtrate was transferred to a 250-mL separatory funnel containing 15 g of NaCl, which was stirred at 250 rpm for 5 min. The acetonitrile extract was allowed to flow through an anhydrous sodium sulfate layer for dehydration. Subsequently, 20 mL of extract was collected and rotary-evaporated to dryness at 40°C; the obtained residue was dissolved in 10 mL of n-hexane. Florisil (5 g) that was activated for at least 5 h at 130°C was wet filled into a glass column, and ~2 g of anhydrous sodium sulfate was added on top of the upper layer of florisil. The column was subsequently washed with 50 mL of hexane and stabilized. The abovementioned n-hexane extract (10 mL) was added to the top of column and allowed to flow through; in addition, 40 mL of n-hexane employed for washing the container was also allowed to continuously flow through the column. Etofenprox was subsequently eluted using 40 mL of a hexane:acetone (95:5, v/v) mixed solvent, and subsequently rotary-evaporated at 40°C in a water bath. The concentrated and dried samples were redissolved in 2 mL of acetonitrile.

The residual pesticide was analyzed using an HPLC apparatus (Shiseido SI2, Tokyo, Japan) coupled with a UV detector (Shiseido, Tokyo, Japan). The HPLC setup was equipped with a SunFire column (length of 250 mm, I.D. of 4.6 mm, 5 μm; Waters, Leinster, Ireland) and run at 40°C. A wavelength of 225 nm, and an injection volume and flow rate of 20 μL and 1.0 mL/min, respectively, were employed. Acetonitrile and deionized water were used as mobile phases A and B, respectively. The gradient was programmed as follows: 0, 3, 10, 16, 17, 30, 31, and 40 min corresponded to 60, 60, 95, 95, 100, 100, 60, and 60% of A, respectively.

### Rice processing methods

#### Rice cakes

Polished rice (1.7 kg) was washed three times with water and soaked in 2.5 L of water for 12 h; the rice mixture was subsequently placed in a 20-mesh sieve for 30 min to facilitate water removal. Salt (17 g) and sugar (170 g) were added to this mixture, which was subsequently ground three times using a rice grinder (Duksan food industry, Chungbuk, Republic of Korea); the resulting rice flour was filtered using the 20-mesh sieve. Water (170 mL) was added to the sieved rice flour, which was placed in a steamer pot, steamed for 20 min (HY-2009-A®, Hanyangmetal, Ulsan, Republic of Korea), and left to cool to room temperature (20–25°C) for 10 min. To comparatively evaluate the changes in pesticide residues during the preparation of rice cakes, samples of polished rice, polished rice immersed in water for 12 h, rice flour, rice flour mixed with water, and rice cakes were collected for analysis.

To analyze the effect of heat treatment on the removal of pesticide residues, rice cakes with unwashed polished rice were prepared with heating durations of 10, 15, 20, and 25 min. Samples obtained from each heating experiment were collected to facilitate a comparative analysis of the changes in pesticide residue levels during the heating process.

#### Rice cookies

Polished rice (1.2 kg) was washed three times with water and immersed in water (1.8 L) for 12 h; the rice mixture was subsequently placed in a 20-mesh sieve for 30 min to facilitate water removal. Sugar (77 g), salt (11 g), and baking powder (33 g) were added to the rice, and the mixture was subsequently ground three times in the rice grinder; the resulting mixture was sieved. Margarine and the obtained rice flour were placed in a dough kneader to ensure uniform mixing; warm water (660 mL) was also added to facilitate the mixing process. The container with prepared dough was sealed and placed in a refrigerator for 30 min. Subsequently, two types of rice cookies were prepared using this dough: the first set was obtained by baking in an oven (Phantom electric deck oven®, Sam Jung, Seoul, Republic of Korea), while the other was obtained by frying with cooking oil (Soybean oil®, CJ, Seoul, Republic of Korea). The temperatures of the top and bottom zones in the oven were 180 and 160°C, respectively, and the cookies were baked for 20 min. Fried cookies were prepared using cooking oil that was maintained at 170°C. Finally, samples of polished rice, polished rice immersed in water for 12 h, rice flour, dough, baked cookies, and fried cookies were collected for comparative analysis of the changes in pesticide residue levels in each stage of the cookie preparation.

To analyze the effect of oven-based heat treatment on the reduction in pesticide residue levels during the processing of rice into baked cookies, unwashed polished rice was selected and heated in the oven for durations of 10, 15, and 20 min. Samples obtained from each heating experiment were collected to facilitate a comparative analysis of the changes in pesticide residue levels during oven heating.

### Validation of the analytical method

The linearity of calibration curves at concentrations of 0.05–5 mg/L was assessed using the coefficients of determination (R^2^). To verify the reproducibility of the testing equipment, 0.3 mg/L of the standard etofenprox solution was injected 10 times into the HPLC apparatus, and the retention time (RT) on the chromatogram and variations in peak area were comparatively examined. The limit of detection (LOD) and limit of quantification (LOQ) were calculated using signal-to-noise ratios of 3 and 10, respectively, compared to the background noise of a blank sample. Recovery experiments were carried out with blank samples to validate the analytical method proposed for etofenprox residues. The acceptance criterion for the coefficient of variation(CV) was ≤20%. Prior to extraction, the polished rice sample was treated with the pesticide at four different concentrations: LOQ (0.03 mg/kg), 10 × LOQ (0.3 mg/kg), 50 × LOQ (1.5 mg/kg), and the highest pesticide concentration in the sample (3 mg/kg); three different concentrations of LOQ, 10 × LOQ, and 50 × LOQ were employed for the rice products.

### Statistical analysis

The obtained data were statistically evaluated by one-way analysis of variance (ANOVA) using the Statistical Analysis System software (SAS version 9.1). The Duncan’s multiple range test was employed when significant differences were found (p < 0.05) to determine the differences among means.

## Results and discussion

### Linearity and reproducibility

The linearity of calibration curves was acceptable with correlation coefficients >0.999, and errors generated between repeated analyses were insignificant. Therefore, the stability and reproducibility of the analytical method presented in this study were confirmed.

### LOQ and recovery

The LOD and LOQ used for the analysis of etofenprox were 0.015 and 0.03 mg/kg, respectively. Samples of brown rice, polished rice, and processed rice products, in which the pesticide residues were not detected, were treated with the reference standard solutions of etofenprox at concentrations (fortifications) of 0.03, 0.3, 1.5 and 3 mg/kg. The results of the analysis of etofenprox recovery rates are listed in Table 2. The recovery rates are 85.7–100.3%, 86.2–94.1%, and 91.6–101.9%, at etofenprox concentrations of 0.03, 0.3 and 1.5 mg/kg, respectively, in each of the processed rice products, and 101.1% at 3 mg/kg in polished rice. The average recoveries (%) for all the analytes are within the range of 85.7–101.9% with repeatability and reproducibility below 8.4%, indicating that the repeatability of the method is sufficiently reliable. Therefore, the recovery rates obtained in this study satisfy the Codex guidelines, which are internationally accepted for pesticide analysis [24], regardless of the level of processing and types of the analyzed processed products.

**Table 2:** Recoveries of etofenprox in rice samples and processed rice products (n = 3).

Sample	Fortification with etofenprox (mg/kg)	Recovery Mean ± SD^a^	CV^b^	LOQ^c^ (mg/kg)
**Polished rice**	0.03	88.7 ± 6.2	7.0	0.03
	0.3	92.1 ± 2.5	2.7	
	1.5	94.2 ± 4.4	4.5	
	3.0	101.1 ± 2.3	2.3	
**Rice cakes**	0.03	92.4 ± 1.5	1.7	
	0.3	93.0 ± 5.1	5.5	
	1.5	101.9 ± 4.1	4.0	
**Rice cookie dough**	0.03	85.7 ± 3.8	4.5	
	0.3	94.1 ± 5.7	6.0	
	1.5	91.6 ± 2.1	2.3	
**Oven-baked rice cookies**	0.03	100.3 ± 4.8	4.8	
	0.3	86.2 ± 7.2	8.4	
	1.5	99.6 ± 4.7	4.7	
**Fried rice cookies**	0.03	96.5 ± 1.5	1.5	
	0.3	86.6 ± 6.2	7.2	
	1.5	94.5 ± 5.0	5.3	

^a^SD: standard deviation

^b^CV: coefficient of variation

^c^LOQ: limit of quantification.

### Residual characteristics of etofenprox in rice products

#### Rice cakes

**Fig 1:**
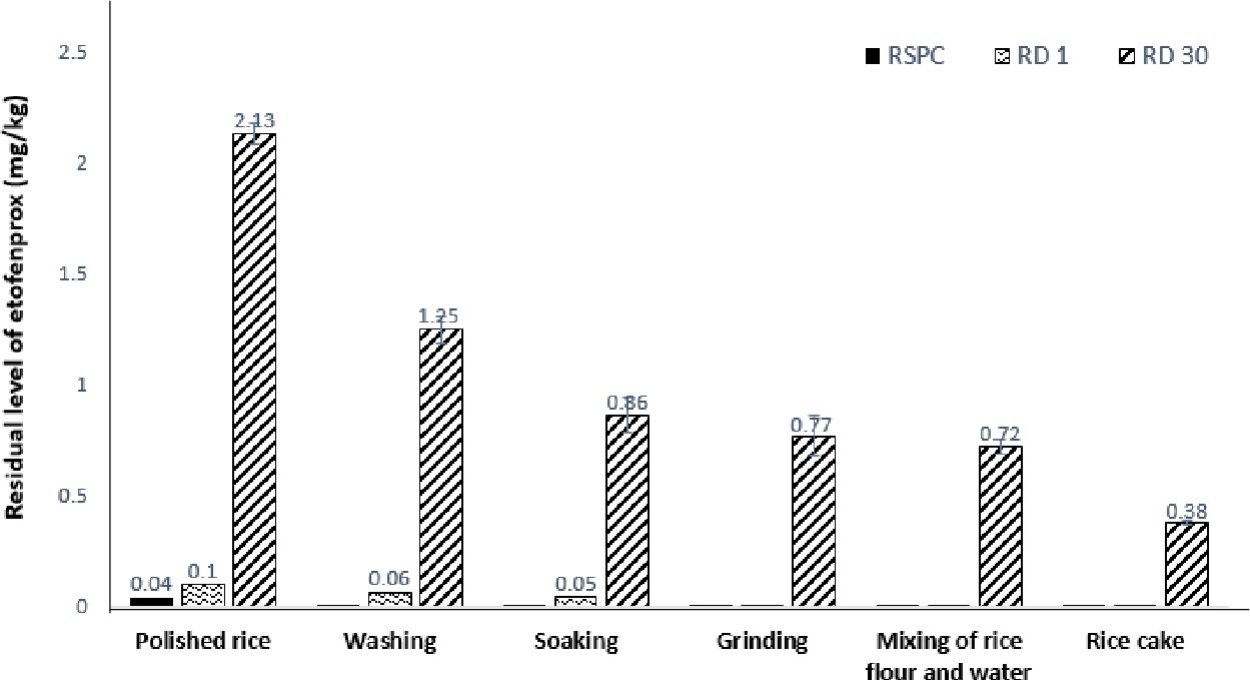
Residual characteristics of etofenprox in the various processing stages of rice cake production.

The residual amount of etofenprox in RSPC (polished rice) was 0.04 mg/kg; however, the pesticide residues were removed after the polished rice was washed, resulting in the absence of pesticide residues in samples obtained via soaking, grinding, and heating during the preparation of rice cakes. The results shown in Fig 1 reveal that the etofenprox residue level in the RD 1 polished rice is 0.1 mg/kg, which decreases to 0.06 mg/kg after washing and to 0.05 mg/kg after soaking of the washed/polished rice in water for 12 h. The results also indicated the removal of pesticide residues in samples that underwent the subsequent grinding and heating processes. Because it was not possible to determine the residual characteristics of etofenprox during the preparation of rice cakes using RSPC and RD 1, RD 30 samples (brown rice dipped in etofenprox solutions) were therefore selected for investigating the residual characteristics of etofenprox in the various stages of rice processing.

The residue level of etofenprox in polished rice for the preparation of rice cakes is noted to be 2.13 mg/kg, which decreases to 1.25 mg/kg (a decrease of 41.3%) after washing. The residue level decreases further to 0.86 mg/kg (a decrease of 59.6%) after soaking of the sample for 12 h, followed by a decrease to 0.77 mg/kg (a reduction of 63.8%) during the preparation of rice flour by grinding and subsequent use of the 20-mesh sieve. The addition of water to rice flour results in a residue level of 0.72 mg/kg (a decrease of 66.4%), and finally a residue level of 0.38 mg/kg (a decrease of 82.1%) after the heating of rice flour with water for 20 min. It should be noted that washing of rice results in a reduction of 41.3% in the etofenprox residues. Kim et al. [27] reported that the residual amount of phenthoate in rice decreased by 51% after the rice was washed three times. In addition, Han et al. [28] reported that the residual levels of chlorpyrifos-methyl, pirimiphos-methyl, fenitrothion, fenthion, and phenthoate in polished rice decreased by 54.2, 47.5, 50.7, 61.5, and 53.4%, respectively, after the polished rice samples were washed. Therefore, the post-washing pesticide reduction rates of etofenprox obtained in this study were similar to those obtained in previous studies.

The processing of unwashed/polished rice was also studied to examine the residual characteristics of etofenprox in terms of heating durations. Table 3 reveals that the etofenprox residue levels are 0.58, 0.54, 0.46, and 0.44 mg/kg after 10, 15, 20, and 25 min of heating, respectively. With an increase in the heating time, the removal rates of residual etofenprox are observed to be 72.9, 74.6, 78.3, and 79.5%, respectively, confirming the decrease in residual etofenprox levels. Several studies have investigated processing methods similar to the heating process employed herein for the preparation of rice cakes. Han et al. [28] reported that captan was not detected in cooked rice, and the residue levels of carbaryl, chlorpyriphos methyl, pirimiphos-methyl, fenitrothion, fenthion, and phenthoate in cooked rice decreased by 91, 61.5, 53.4, 54.5, 47.5, and 50.7%, respectively. Typically, vapor pressures of pesticides have been employed to explain pesticide reduction rates during food processing. The vapor pressures of captan and carbaryl (1.3 mPa (25°C) and 4.1 × 10^−2^ mPa (23.5°C), respectively) are higher than that of etofenprox (8.13 × 10^−4^ mPa at 20°C). This difference in vapor pressure is presumably due to the captan and carbaryl residues in rice exhibiting higher reduction rates after cooking [28] than that of etofenprox (82.1%) in this study. Similarly, the other pesticides analyzed by Han et al. [28] were thought to have a lower pesticide reduction rate than that of etofenprox owing to their lower vapor pressures (and low volatility) than that of etofenprox. By contrast, Hwang et al. [29] reported that the residual levels of isoprothiolane and phthalide in cooked rice decreased by 66.0% and 79.0%, respectively. The vapor pressures of isoprothiolane and phthalide are 4.93 × 10^−1^ mPa (25°C) and 3 × 10^−3^ mPa (23°C), respectively, implying that they possess higher volatilities than that of etofenprox. Nevertheless, Hwang et al. [29] reported lower reduction rates than that of etofenprox obtained in this study. The results reported by Hwang et al. [29] agree with those reported by Im et al. [30], suggesting that the reduction rates of pesticide residues can be determined not by the degree of volatility, which is a physiochemical property of pesticides, but by the differences in processing methods. Jegal et al. [31] reported that the residual levels of diazinon, fenitrothion, phenthoate, and O-ethyl O-(4-nitrophenyl) phenylphosphonothioate (EPN) in cooked rice decreased by 77.8, 74.4, 74.0, and 74.0%, respectively; these results are similar to those obtained in the present study.

**Table 3:** Residual characteristics of rice cakes processed with unwashed polished rice (n = 3).

Sample	Processing method	Residual level (mg/kg) Mean ± SD	% Loss^a^
**RD 30**	Polishing	2.13 ± 0.05a	-
	Soaking	1.29 ± 0.09b	39.6
	Grinding	1.17 ± 0.02c	44.9
	Mixing of rice flour and water	1.10 ± 0.03c	48.2
	Heating for 10 min	0.58 ± 0.005d	72.9
	Heating for 15 min	0.54 ± 0.05d	74.6
	Heating for 20 min	0.46 ± 0.04e	78.3
	Heating for 25 min	0.44 ± 0.02e	79.5

^a^((Raw product residue − processing residue)/Raw product residue) × 100

Values followed by the same letter in the same column are not significantly different (p < 0.05).

#### Rice cookies

Table 4 lists the residual characteristics of etofenprox in terms of the processing stages of oven-baked and fried cookies. Similar to the experiments on rice cakes, the residual properties of etofenprox in the RSPC and RD 1 samples during cookie processing could not be determined. The etofenprox residue level during the preparation of rice cookies with RD 30 in the form of rice flour, which was obtained by grinding and sieving, is noted to be 0.77 mg/kg (a decrease of 63.8%); the etofenprox residue level in the RD 30 rice dough is 0.29 mg/kg (a decrease of 86.3%). The etofenprox residue levels in the baked and fried rice cookies are observed to be 0.47 mg/kg (a decrease of 78.0%) and 0.18 mg/kg (a decrease of 90.2%), respectively. Notably, the etofenprox residues decrease by 63.8% in rice flour obtained via the grinding of polished rice. However, Park et al. [32] reported that the residual amounts of azinphos-methyl, chlorpyrifos, chlorpyrifos-methyl, fenitrothion, malathion, and trichlorfon decreased by 95, 94, 95, 93, 93, and 94%, respectively; all these reductions were higher than those obtained in this study. The reduction in etofenprox residues in rice flour obtained by grinding of washed/polished rice was focused on in the present study; however, Park et al. [32] investigated the reduction rate of pesticide residues after removing the rice husks and the foreign matter around the husks via tempering, crushing, and grinding, a process which is similar to the milling process of wheat [33]. The use of a different milling process presumably contributed to the higher reduction rates of pesticide residues in the study by Park et al. In this study, the processing of rice flour into baked rice cookies reduced the residual levels of etofenprox by 38.9% (from 0.77 to 0.47 mg/kg). In previous studies on the reduction of pesticide residues in wheat flour-based oven-baked bread, the residual levels of pirimiphos-methyl, fluxapyroxad, and azoxystrobin were reported to decrease by 48% [34], 40% [35], and 40% [36], respectively, the similar to decrease in etofenprox residue level during the heating stage of rice cookie production in this study.

**Table 4:** Residual characteristics of etofenprox in the processing stages for baked and fried rice cookies (n = 3).

Sample	Processing method	Residual level (mg/kg) Mean ± SD	% Loss
**RSPC**	Polishing	0.04 ± 0.001	-
	Washing	ND	100
	Soaking	ND	100
	Grinding	ND	100
	Dough mixing	ND	100
	Baking	ND	100
	Frying	ND	100
**RD 1**	Polishing	0.10 ± 0.005	-
	Washing	0.06 ± 0.002	40.0
	Soaking	0.05 ± 0.003	50.0
	Grinding	ND	100
	Dough mixing	ND	100
	Baking	ND	100
	Frying	ND	100
**RD 30**	Polishing	2.13 ± 0.05a	-
	Washing	1.25 ± 0.06b	41.3
	Soaking	0.86 ± 0.08c	59.7
	Grinding	0.77 ± 0.09c	63.8
	Dough mixing	0.29 ± 0.04d	86.3
	Baking	0.47 ± 0.10e	78.0
	Frying	0.21 ± 0.02e	90.2

ND: not detected

Values followed by the same letter in the same column are not significantly different (p < 0.05)

The effect of heating duration on the reduction in residual levels of etofenprox were examined using baked and fried cookies prepared using unwashed polished rice. This experiment was performed to confirm the thermal decomposition tendency of pesticides with respect to heating time if rice washing was insufficient. Table 5 shows that the residual amount of etofenprox in the dough is 0.58 mg/kg, and those in the rice cookies baked in the oven for 10, 15, and 20 min are 0.61, 0.77, and 1.05 mg/kg, respectively. The residual etofenprox level is noted to increase in rice cookies with heating durations of 15 and 20 min compared to that of the dough; this is possibly because of the decrease in moisture content during oven baking of the dough (moisture content reduces from 42.16 to 5.55%), which can enhance the concentration of etofenprox. However if the effect of the moisture content reduction is considered, the pesticide residue is clearly decreased.

**Table 5:** Residual characteristics of baked and fried rice cookies prepared using unwashed polished rice (n = 3).

Sample	Processing method	Residual level (mg/kg) Mean ± SD	% Loss	Moisture content (%)
**RD 30**	Polishing	2.13 ± 0.05a	-	12.62
	Soaking	1.29 ± 0.08b	39.6	33.76
	Grinding	1.17 ± 0.02c	44.9	33.64
	Dough mixing	0.58 ± 0.02f	72.7	42.16
	Baking for 10 min	0.61 ± 0.06f	71.7	22.29
	Baking for 15 min	0.77 ± 0.04e	63.7	15.57
	Baking for 20 min	1.05 ± 0.07d	50.9	5.55
	Frying	0.37 ± 0.03g	82.6	11.28

Values followed by the same letter in the same column are not significantly different (p < 0.05)

The etofenprox residue levels in rice cookies fried and baked at 180°C are noted to be 0.37 mg/kg (a decrease of 82.6%) and 1.05 mg/kg (a decrease of 50.9%), respectively. Therefore, the frying process is considered to have a better effect than baking on the reduction of pesticide residues. The octanol–water partition coefficient (K_OW_) is 6.9 for etofenprox. If K_OW_ < 3.3, the pesticide is classified as water-soluble, and if K_OW_ > 3.3, the pesticide is classified as fat-soluble [37]. Etofenprox is classified as a strongly fat-soluble pesticide according to this guideline and is presumed to be transferred to the oil during the frying stage in the preparation of fried rice cookies. Similar results have been obtained in other studies as well. The residual amounts of pesticides in potatoes treated with imazalil were reported to decrease by 51 and 98% after the baking and frying process, respectively [38]. In addition, the residual levels of fluxapyroxad and pydiflumetofen after baking were found to decrease by 50 and 55%, respectively [39, 40]. Furthermore, the residual levels of carbaryl and difenoconazole were reported to decrease by 96 and 92%, respectively, after the frying process [41, 42].

## Conclusions

This study was conducted to obtain residual data of pesticides in states prior to food intake for realistic exposure evaluation of pesticide residues in rice cakes and cookies. Etofenprox was not detected after washing the polished sample of RSPC, and the tendency of decreasing pesticide levels during rice processing into cakes and cookies could not be determined. This suggested that etofenprox could be sufficiently removed during processing even if it was sprayed at a dose higher than the recommended safe dose. In the case of RD 1, although the etofenprox was dipped in brown rice at the MRL, it was not detected after grinding. The high-concentration RD 30 sample was used to investigate the residual characteristics of etofenprox in the various stages of rice processing; decreases in pesticide levels of 82.1, 78.0, and 90.2% were observed in rice cakes, baked rice cookies, and fried rice cookies, respectively. These reductions in the etofenprox residues were presumed to be caused by washing, volatilization, and thermal decomposition. In conclusion, the removal of etofenprox residues below the MRL can be easily facilitated by washing, milling, and heat processing of rice.

## Supporting information

S1 Fig Standard calibration curves for the quantitation of etofenprox. (TIF)

S2 Fig Chromatogram of etofenprox standard. (TIF)
