# Correction: Residual characteristics of etofenprox in the processing stages of rice cakes and cookies

**DOI:** 10.1371/journal.pone.0272391

**Published:** 2022-07-28

**Authors:** HyeSu Lee, Moo-Hyeog Im

In the Funding section, the grant number from the funder Ministry of Food and Drug Safety in 2019 is listed incorrectly. The correct grant number is: 19162MFDS021.
