# Supplementary figures and images for: Residual characteristics of etofenprox in the processing stages of rice cakes and cookies
Source: PLoS One. 2021 Aug 6;16(8):e0255751. doi: 10.1371/journal.pone.0255751 (PMC8345890; doi:10.1371/journal.pone.0255751)

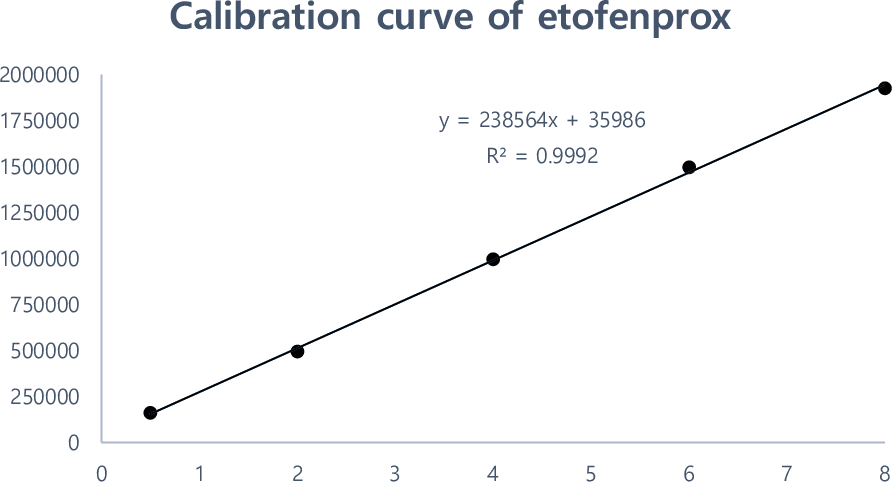

Supplement: S1 Fig — (TIF) [file pone.0255751.s001.tif]

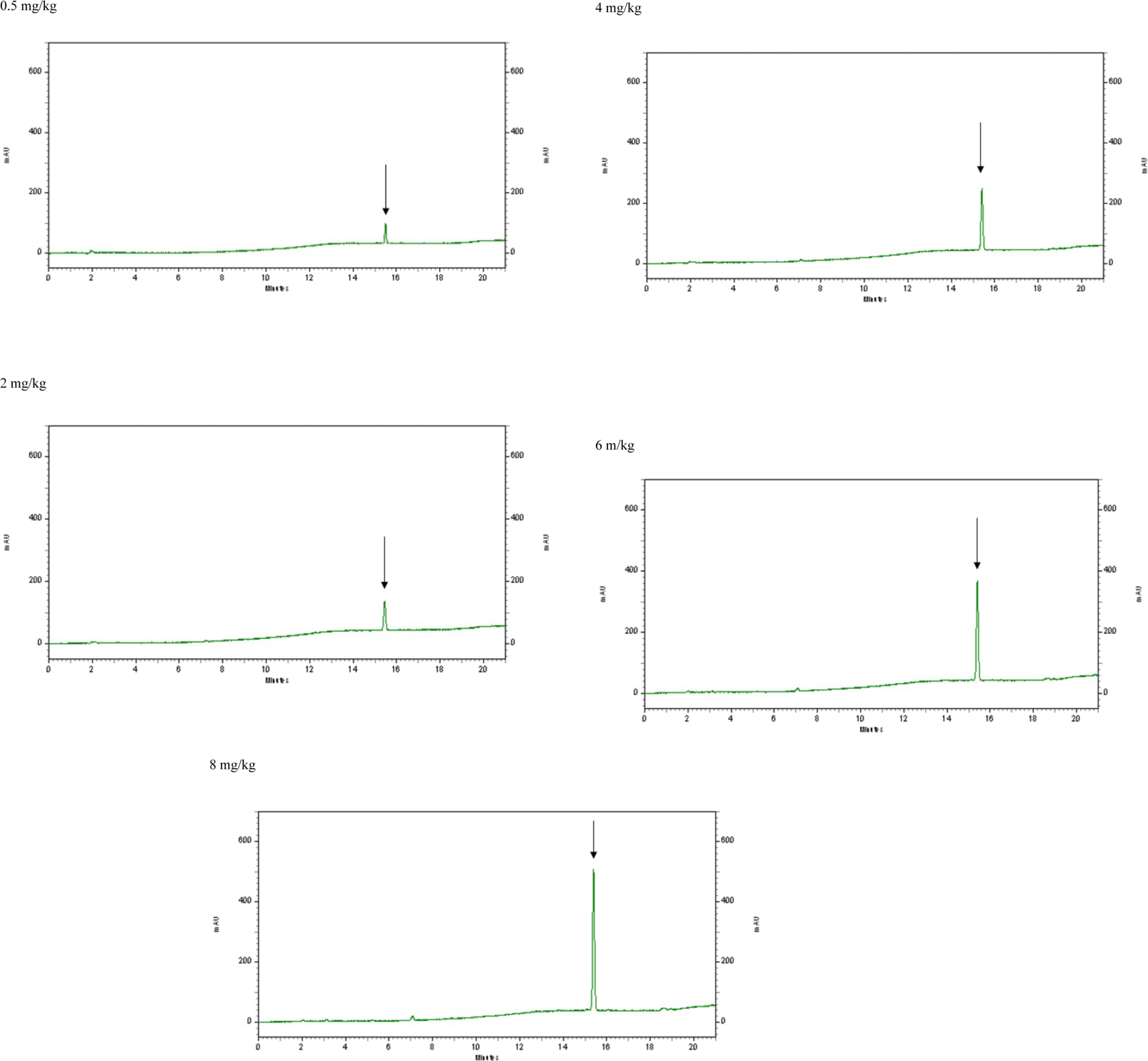

Supplement: S2 Fig — (TIF) [file pone.0255751.s002.tif]
